# Microbial succession and its effect on the formation of umami peptides during sufu fermentation

**DOI:** 10.3389/fmicb.2023.1181588

**Published:** 2023-04-17

**Authors:** Jieqi Mao, Zhilei Zhou, Hongshun Yang

**Affiliations:** ^1^Department of Food Science and Technology, National University of Singapore, Singapore, Singapore; ^2^National Engineering Research Center of Cereal Fermentation and Food Biomanufacturing, School of Food Science and Technology, Jiangnan University, Wuxi, Jiangsu, China; ^3^Shaoxing Key Laboratory of Traditional Fermentation Food and Human Health, Jiangnan University (Shaoxing) Industrial Technology Research Institute, Shaoxing, Zhejiang, China

**Keywords:** sufu, solid-state fermentation, umami peptide, microbiome, interaction network

## Abstract

Sufu, a traditional Chinese fermented food, is famous for its unique flavor, especially umami. However, the formation mechanism of its umami peptides is still unclear. Here, we investigated the dynamic change of both umami peptides and microbial communities during sufu production. Based on peptidomic analysis, 9081 key differential peptides were identified, which mainly involved in amino acid transport and metabolism, peptidase activity and hydrolase activity. Twenty-six high-quality umami peptides with ascending trend were recognized by machine learning methods and Fuzzy c-means clustering. Then, through correlation analysis, five bacterial species (*Enterococcus italicus*, *Leuconostoc citreum*, *L. mesenteroides*, *L. pseudomesenteroides*, *Tetragenococcus halophilus*) and two fungi species (*Cladosporium colombiae*, *Hannaella oryzae*) were identified to be the core functional microorganisms for umami peptides formation. Functional annotation of five lactic acid bacteria indicated their important functions to be carbohydrate metabolism, amino acid metabolism and nucleotide metabolism, which proved their umami peptides production ability. Overall, our results enhanced the understanding of microbial communities and the formation mechanism of umami peptides in sufu, providing novel insights for quality control and flavor improvement of tofu products.

## 1. Introduction

Food-derived peptides exhibit various functions, such as antihypertension, antimicrobial, and antioxidant, and may also contribute to the taste of food (Raikos and Dassios, 2014; Kamran and Reddy, 2018). Umami, which is described as a pleasant savory taste, is the fifth basic taste aside from sweet, sour, salty, and bitter (Yamaguchi and Ninomiya, 1998; Lindemann et al., 2002). Umami plays an important role in food flavor, and many umami ingredients such as monosodium glutamate (MSG), 5′-inosine monophosphate (IMP), and 5′-guanosine monophosphate (GMP) (Yamaguchi, 1967; Ault, 2004; Zhang et al., 2013) are widely used in food seasoning. After the first discovery of umami peptides from soybean protein hydrolyzates (Arai et al., 1972), umami peptides have attracted extensive attention from scientists (Zhang et al., 2017, 2019). Besides adding umami to the food, umami peptides can also modulate the overall flavor through a synergistic effect via taste receptors (Shim et al., 2015; Dang et al., 2019; Wang et al., 2020; Xie et al., 2023). Umami peptides generally have a molecular weight of less than 3000 Da (Yu et al., 2017; Zhang et al., 2019; Liu et al., 2020), and those with a molecular weight between 500 and 1000 Da are assumed to have the strongest umami taste (Rhyu and Kim, 2011). Fermentation is an effective way to create umami peptides (Zhao et al., 2016, 2019). During fermentation, protein and large peptides are hydrolyzed by proteases through microbial metabolism to form abundant small peptides. Umami peptides have been identified from many traditional fermented foods, including soy sauce (Lioe et al., 2006; Zhuang et al., 2016), fish sauce (Bu et al., 2021; Zhu et al., 2021), and ham (Dang et al., 2015; Xie et al., 2023). Recently, researchers also discovered umami peptides in sufu, and their salt-enhancing effect had been proven (Xie et al., 2018a; Chen et al., 2021).

Sufu, also called fermented soybean curd or furu, is a traditional Chinese dish with a long history of over a thousand years (Han et al., 2001b). Sufu is widely consumed as a condiment due to its mellow texture, special fragrance, and umami flavor (Han et al., 2004a; Cai et al., 2021; Liu et al., 2022). Made by fermentation of tofu, sufu also has very rich nutritional value, especially high protein content of about 40% (dry matter basis) (Han et al., 2004b). Microorganisms is believed to be of vital importance to the quality of sufu. During fermentation, various kinds of enzymes produced by microbial communities hydrolyze carbohydrate, lipid and especially proteins in soybeans into small molecules, which are more volatile, bioactive and easy to digest (Xu et al., 2016; Cai et al., 2021; Li et al., 2022). Although most sufu products are fermented with a pure culture starter, bacterial and fungal communities from the environment also play a non-negligible role in sufu fermentation due to the open production environment (Han et al., 2001a, 2004a; Cai et al., 2016). Studies have been conducted on the diversity of environmental microorganisms based on geography, production methods, and fermentation stages (Han et al., 2004a; Cai et al., 2021). And the correlation between microorganisms and volatile flavor components in sufu is also well studied (Xie et al., 2018b; He and Chung, 2020; Li et al., 2022). However, there is still little knowledge of how microorganisms contribute to the formation of umami peptides during fermentation.

Until now, about 208 umami peptides have been reported. While most research on umami peptides focused on isolation and identification, their formation mechanism is still unclear. With the development of high-throughput sequencing technology, using peptidomics and microbiomics to discover new umami peptides and to study the mechanism of microorganisms and umami peptides are of great value for subsequent industrial applications. The study on peptidomics throughout sufu fermentation is relatively lacking. And the research on the changes of endogenous polypeptides as well as the function of precursor proteins during the whole fermentation process is still blank. Also, studies on fermenting microorganisms in sufu are mainly at the genus level rather than species level. Based on these facts, 24 sufu samples at different fermentation stages were collected in this study. First, the endogenous peptides during the whole fermentation process were identified. The key functional peptides were filtered, and the function of their precursor proteins was analyzed. Then, high-quality potential umami peptides were identified based on multiple machine-learning methods. The structural characteristics and diversity of the microbial community during sufu fermentation were determined by full-length amplicon sequencing. Finally, the microbial strains significantly correlated with umami peptides were identified. Overall, this study provides a theoretical reference for mining key microorganisms that determine or regulate the entire fermentation process of sufu, and provides guidance for the development and industrial application of umami peptides.

## 2. Materials and methods

### 2.1. Sampling and sample treatment

**FIGURE 1:**
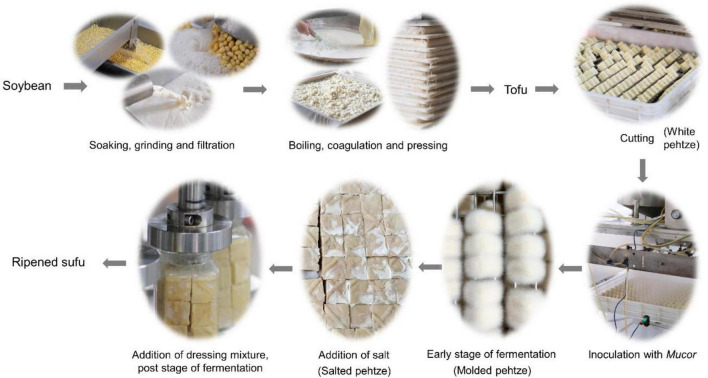
Fermentation process of sufu.

Fresh white sufu samples at different fermentation stages were harvested at a representative sufu factory located at Shaoxing, Zhejiang Province, China (120°32′E, 31°51′N). The manufacturing process of white sufu is shown in Figure 1. Soybeans were first washed and soaked in water, then grinded to obtain soymilk. Then, the filtered soymilk was boiled and coagulated by adding gesso, and pressed to make tofu. After cutting into small cubes, the diced tofu, also called white pehtze, was inoculated with *Mucor* spp. and fermented at 25–30°C for about 25 h to allow full growth of hypha. Subsequently, the molded pehtze was marinated by salt for about 2 days. Finally, the salted pehtze was post-aged in closed bottles after adding a dressing mixture containing edible alcohol until ripening. The samples of white pehtze (WP), molded pehtze after inoculating for 12 and 25 h (P12, P25), salted pehtze (SP), posted-fermented sufu at 7, 30, 60, 90 days (F7, F30, F60, F90) were collected for analysis. Nine bottles of samples were randomly collected at different stages as described above, and samples from each three bottles were mixed into one sample. All samples were stored at −80°C until further analysis.

### 2.2. Free peptide determination by peptidomics analysis

#### 2.2.1. Peptide extraction

Sufu samples were ground in liquid nitrogen, and then mixed with methanol, chloroform and water at a ratio of 3:1:4 (V: V). After a brief vortex, the mixture was centrifuged at 4700 rpm for 10 min at 4°C. The upper aqueous supernatant was collected. Then, the collected supernatant was desalted using Waters Oasis MCX desalination column (Waters, Milford, MA, USA). The flow-through was collected and lyophilized.

#### 2.2.2. Identification by LC-MS/MS

The lyophilized sample was redissolved in distilled water with 0.1% formic acid for 10 μg/μL. The sample was analyzed using Nanoflow LC-MS/MS on a quadrupole Orbitrap mass spectrometer (Q Exactive HF-X, Thermo Fisher Scientific, Bremen, Germany) coupled to an EASY nLC 1200 ultra-high-pressure system (Thermo Fisher Scientific) via a nano-electrospray ion source. Peptides were separated using a gradient from 8 to 12% B in 5 min, then 12–30% B in 33 min and stepped up to 40% in 7 min followed by a 15 min wash at 95% B with a flow rate of 0.60 μL/min onto the Thermo C-18 column (150 μm × 250 mm, Thermo, Waltham, MA, USA), where solvent A was 0.1% formic acid in water and solvent B was 80% ACN and 0.1% formic acid in water. The total duration of the run was 60 min, and the column temperature was kept at 60°C. Briefly, the mass spectrometer was operated in “top-40” data-dependent mode, collecting MS spectra in the Orbitrap mass analyzer (120,000 resolution, 100–1500 m/z range) with an automatic gain control (AGC) target of 3E6 and a maximum ion injection time of 80 ms. The most intense ions from the full scan were isolated with an isolation width of 1.6 m/z. Following higher-energy collisional dissociation (HCD) with a normalized collision energy (NCE) of 27, MS/MS spectra were collected in the Orbitrap (15,000 resolution) with an AGC target of 5E4 and a maximum ion injection time of 45 ms. Precursor dynamic exclusion was enabled with a duration of 16 s. All RAW files were analyzed using PEAKS (version 8, Bioinformatics Solutions Inc.) (Ma et al., 2003). MS2 spectra collected in the Orbitrap was searched against the Glycine max (protein number: 74,863, database: uniport, download time: 2022.09.15) with parameters as following: precursor mass tolerance of 15 ppm and a fragment mass tolerance of 0.02 Da; ALC (%) setting greater than 50%; false discovery rate (FDR) of less than 1% (Tran et al., 2019).

#### 2.2.3. Identification of differential peptides and function analysis of precursor protein

Based on the quantitative results, *T*-test was used for differential analysis between each group and the unfermented group WP, respectively. Differential peptides were screened based on the standard | log2(FoldChange)| > 1 and false discovery rate (FDR) < 0.05. The function of precursor protein was annotated using COG (Eukaryotic Orthologous Groups) protein orthologous cluster database,^1^ SwissPro protein sequence database,^2^ KEGG (Kyoto Encyclopedia of Genes and Genomes) Kyoto Gene Encyclopedia Database,^3^ GO (Gene Ontology) Gene Ontology Database.^4^ KEGG and GO functional enrichment analysis were performed using R studio.

#### 2.2.4. Screening of umami peptides

In order to improve the efficiency, three machine learning methods iUmami-SCM,^5^ UMPred-FRL^6^, and Umami_YYDS^7^ were used for screening of umami peptides. All the three databases predicted umami peptides mainly based on their primary sequence information. However, iUmami-SCM database used a scoring card method (SCM) (Charoenkwan et al., 2020), while UMPred-FRL used a feature representation learning method (FRL) (Charoenkwan et al., 2021). Umami_YYDS built a larger database by collecting reported umami peptide information to improve accuracy (Cui et al., 2023). Umami peptides were screened based on three criteria: (a) Peptide length less than 10. (b) Predicted as umami by all three methods. (c) The probability rate should be over 80%.

### 2.3. Amplicon sequencing

#### 2.3.1. Genomic DNA extraction, PCR amplification and sequencing

The metagenomic DNA of samples was extracted using CTAB-based method (Allen et al., 2006). The quality of each extracted DNA was determined by a NanoDrop 2000c Spectrophotometers (Thermo Fisher Scientific, Wilmington, MA, USA) and 1% agarose gel electrophoresis. For bacterial, the full-length 16S rRNA V1–V9 region was amplified by PCR with universal primers 27F (5′-AGGRGTTTGATYNTGGCTCAG-3′) and 1492R (5′-TASGGHTACCTTGTTASGACTT-3′) (Lane, 1991). For fungus, the fungal internal transcribed spacer (ITS) region was amplified with primer sets of ITS1f (5′-CTTGGTTCATTTAGAGGAAGTAA-3′) (Gardes and Bruns, 1993) and ITS4 (5′-TCCTCCGCTTATTGATATGC-3′) (White et al., 1990). Sequencing of both libraries was performed in the Circular Consensus Sequencing (CCS) mode on the PacBio RS II platform (Pacific Biosciences, Menlo Park, CA, USA).

#### 2.3.2. Sequence analysis

Lima (v2.7.1)^8^ was used to identify CCS through barcode, and get raw sequence data. Raw sequence data was corrected and filtered with 99% accuracy and desired sequence length to remove adapter pollution and low-quality data by cutadapt (v1.9.1) (Martin, 2011). UCHIME (v4.2) software was used to identify and remove chimeric sequences to obtain Effective sequences (Edgar, 2016). Based on 97% similarity, UCLUST was used to cluster high-quality sequences into different Operational Taxonomic Units (OTUs) (Edgar, 2010). A single representative sequence from each clustered OTU was used for annotation with the Silva database (v13.2) and the UNITE fungal database (v12.11) to obtain taxonomic information (Quast et al., 2012; Nilsson et al., 2019). Singleton OTUs was discarded before further analysis to eliminate errors. Chao1 richness index and Shannon diversity index were calculated using QIIME2 (v2022.11) (Dunn, 1973; Bolyen et al., 2019). Visualization of microbial community structure composition was provided by Principal Co-ordinates Analysis (PCoA).

## 3. Results and discussion

### 3.1. Peptidomics and umami peptides during sufu production

#### 3.1.1. Peptide identification based on *de novo* sequencing

**FIGURE 2:**
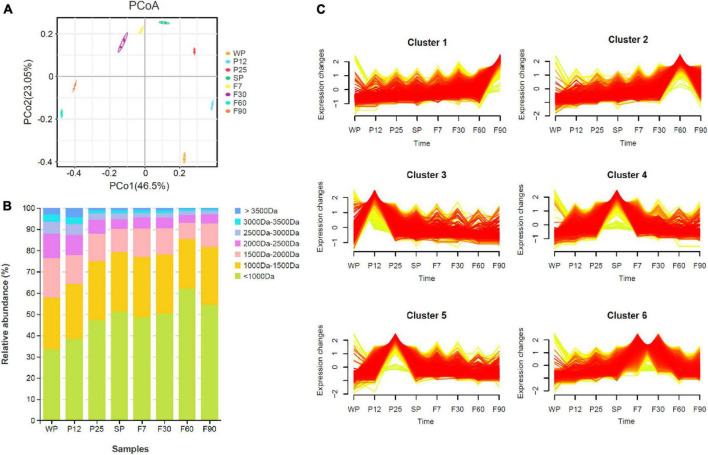
Peptide analysis during fermentation of sufu. **(A)** PCA analysis. **(B)** Molecular weight distribution of total peptides. **(C)** Trend analysis of differentially expressed peptides.

As a branch of proteomics, peptidomics focuses specifically on endogenous peptides present in the sample, and can reflect the process of proteolytic cleavages (Schrader et al., 2014; Dallas et al., 2015). Peptidomics is usually composed of peptide identification and quantitation, function prediction, and high-throughput analysis. Mass spectrometry (MS) is a powerful tool for peptide identification and quantification. In this study, peptidomics composed of MS/MS data analyzed by PEAKS software through *de novo* sequencing and Glycine max database matching was used to quickly identify and characterize peptides from different fermentation stages of sufu. A total of 40,479 peptides were identified in this study at different stages of fermentation. As shown in Figure 2A, principal component analysis (PCA) indicated good parallelism for three biological replicates in each group. The peptides in WP exhibited more difference than other groups, while no significant difference was shown among SP, F7, and F30. The distribution of peptide molecular weight was analyzed in Figure 2B. Peptides with molecular weight less than 1000 Da were dominant during sufu production, and were over 33.3% in all samples. The percentage of peptides less than 1500 Da generally increased through both early and post stage of fermentation. In contrast, peptides with molecular weight greater than 2000 Da displayed a descending tendency in abundance. This phenomenon supported the idea that peptides were hydrolyzed into smaller molecules during sufu fermentation (Xu et al., 2016; Cai et al., 2021; Li et al., 2022).

#### 3.1.2. Identification of differential peptides and function prediction of precursor protein

In order to learn the role of peptides during sufu fermentation, WP was used as the control group to analyze the differential peptides. A total of 9,081 differential peptides were identified in this study. To clarify the changes in endogenous peptides and the functions of precursor proteins throughout the fermentation process, Mfuzz C-Means Clustering was used for temporal trend analysis of differential peptide expression. Mfuzz is able to identify underlying time-series patterns of expression profiles and cluster peptides with similar patterns, which can help us understand the connection between dynamic patterns of peptides and their functions (Dunn, 1973). As shown in Figure 2C, six clusters of peptides with different expression patterns were obtained. Cluster 1 displayed an ascending tendency over time, and includes 937 differential peptides. GO analysis showed that for the most part, the precursor proteins for differential peptides were annotated into the groups of cellular protein catabolic process, cysteine-type endopeptidase activity, peptidase activity, acting on L-amino acid peptides, and hydrolase activity (Supplementary Table 1). The KEGG pathway enrichment analysis was also conducted. The result indicated that the differential peptides were mainly involved in seven different pathways, including carbon metabolism, protein processing in endoplasmic reticulum, glycolysis/gluconeogenesis, biosynthesis of amino acids, and glyoxylate and dicarboxylate metabolism (Supplementary Table 2). COG annotation results show that the main functions of precursor proteins include post translational modification, protein turnover, chaperones, amino acid transport and metabolism, carbohydrate transport and metabolism, secondary metabolites biosynthesis, transport, and catabolism (Supplementary Table 3).

#### 3.1.3. Prediction of umami peptides in sufu

**FIGURE 3:**
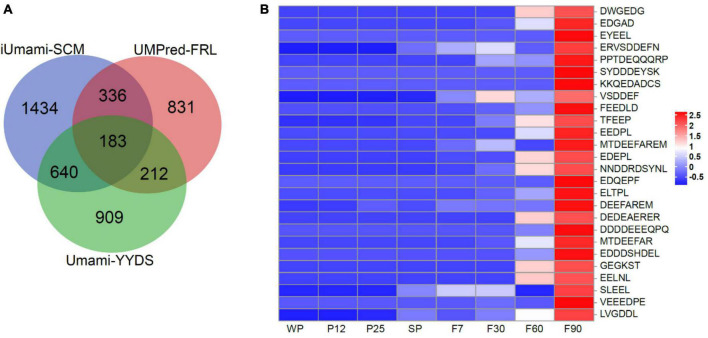
Umami peptides in sufu. **(A)** Venn analysis for the identification of umami peptides. **(B)** Heatmap analysis of umami peptides during fermentation of sufu.

During sufu fermentation, complexes formed by degradation of soybean protein can generate a strong umami taste (Yoshida, 1998; Hajeb and Jinap, 2015). Umami peptides, which have attracted increasing attention, can greatly improve food flavor and enhance taste perception (Zhang et al., 2017, 2019). The study of umami peptides not only provides reference for the quality improvement of food products, but also the development of new seasonings. In this study, three machine learning methods (iUmami-SCM, UMPred-FRL, Umami_YYDS) based on computational techniques were used for rapid screening of large-scale umami peptides (Charoenkwan et al., 2020, 2021; Cui et al., 2023). Among 40,479 endogenous peptides, 3,470, 3,245, and 4,381 peptides were identified as umami by three models respectively (Figure 3A). After filtering and taking the intersection, 183 high-quality candidate umami peptides were obtained. Based on the results of differential peptide trend analysis (Figure 2C), it was surprising to find that 26 umami peptides showed an upward trend consistently in the umami taste during sufu fermentation (Figure 3B). The precursor proteins of these peptides were mainly lipoxygenase, phospholipase D, glycinin G4, and phosphopyruvate hydratase (Supplementary Table 4). Studies had shown that these precursor proteins were related to the formation of umami flavor in food. Overall, the umami peptides we screened had the potential to produce a strong umami taste, which had great significance for the development and application of subsequent umami peptide products.

### 3.2. Microbiota profile during sufu production

#### 3.2.1. Similarity in microbiome composition at different stages

**FIGURE 4:**
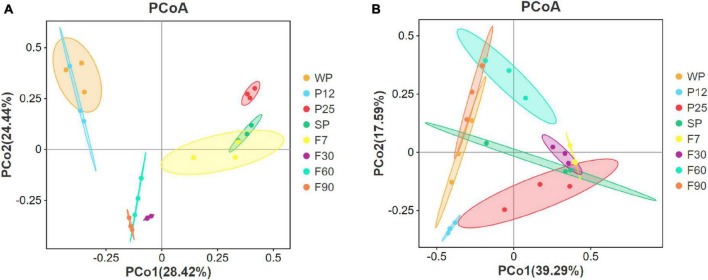
Principal coordinate analysis (PCoA) of the bacterial **(A)** and fungal **(B)** communities based on Bray-Curtis distances.

Microorganisms in sufu metabolize and synthesize flavor substances by secreting various kinds of enzymes. Its community composition plays an important role in regulating the flavor of sufu, and is considered to be a key factor in determining the quality of final products (Tan et al., 2020). In order to evaluate the variation and similarity in microbiome composition at different fermentation stages of sufu, beta diversity was analyzed by principal coordinate analysis (PCoA) plots. As shown in Figure 4, each sufu sample was represented by one dot, and samples in each group were clustered by an ellipse. The closer the two groups were ordinated, the more similar their microbiota profile. The PCoA plot for bacteria indicated the clear difference between WP, P25 and F90 due to their long distance. It was also noticed that the ellipses of WP and P12 were highly overlapped, indicating their high similarity in microbiome composition. Besides, the high similarity was also shown between SP and F7; F60 and F90. During the early stage of fermentation, sufu bacteria community changed the most after inoculation with *Mucor* for 12–25 h. And since the distance between F7 and F30 was longer than that between F30 and F60, F60 and F90, it can be concluded that the bacteria community of sufu mainly changed during the first 30 days rather than the later 60 days. According to Figure 4B, for fungi, P12 and F60 exhibited the most difference in community structures due to their longest distance. F7 and F30 were highly similar due to the large overlap, which was different from the pattern of the bacteria community.

#### 3.2.2. Abundance and diversity of microbial communities

**FIGURE 5:**
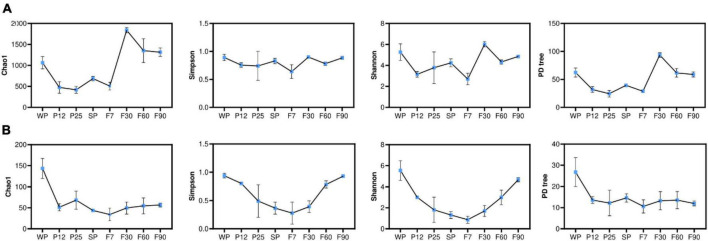
Analysis of alpha diversity index of bacteria **(A)** and fungi **(B)**.

Alpha diversity indices were used to evaluate the abundance and diversity of microbial communities during sufu production (Figure 5). While the Chao1 index and PD tree positively correlated with the abundance or richness of species, Simpson and Shannon index measured the degree of diversity within the community. As shown in Figure 5A, the abundance and diversity of bacterial communities fluctuated over time. It was noticed that Chao1 and PD tree indices significantly increased (*P* < 0.05) from F7 to F30, and continuously decreased after reaching its highest value at F30. Such trend indicated that the abundance of bacterial communities was significantly influenced by the addition of dressing, and was consistent with previous studies (Huang et al., 2018; He and Chung, 2020). The decrease in both abundance and diversity of bacterial communities after ripening for 30 days may due to the depletion of oxygen in the bottle. For fungal communities (Figure 5B), Chao1 and PD tree indices significantly decreased (*P* < 0.05) from WP to P12. These results indicated that the inoculation with *Mucor* significantly decreased the abundance of fungal communities. Simpson and Shannon indices decreased continuously before reaching their lowest value at F7, and kept increasing at the ripening stages. This suggested long ripening time of 90 days contributed to the diversity of fungal communities, which was also supported by previous studies (He and Chung, 2020; Li et al., 2022). It was also noticed that compared with fungal communities, bacterial communities were significantly richer during the whole production process. Such microbial community structure was consistent with other sufu products fermented by *mold*.

#### 3.2.3. Composition of microbial community

**FIGURE 6:**
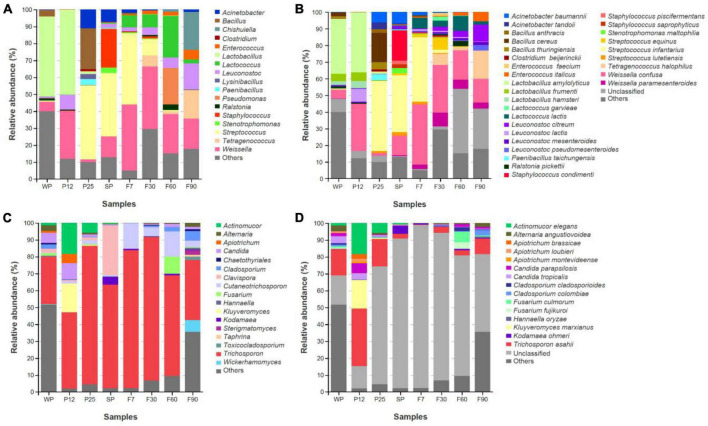
Structure and succession of microbial community during fermentation of bacteria **(A)** and fungi **(C)** at the genus level; structure and succession of microbial community during fermentation of bacteria **(B)** and fungi **(D)** at the species level.

Third-generation sequencing technology has been less studied in the fermentation process of sufu. Compared with second-generation sequencing, full-length sequencing avoids systematic errors due to PCR while improving read length, and therefore restoring the community structure of microorganisms more realistically at the species level (Shin et al., 2016; Callahan et al., 2019; Tedersoo and Anslan, 2019). In order to improve the efficiency and accuracy, full-length ITS and 16S rRNA gene amplicon analysis was used in this study. The composition of bacteria and fungi communities at both the genus and the species levels were compared in Figure 6.

For the bacteria community, a total of 18 genera and 31 species with an abundance greater than 1% were identified from samples at all stages. At the genus level (Figure 6A), the top three bacterial genera across the whole production process were *Weissella*, *Lactobacillus* and *Streptococcus*. Unlike *Weissella*, which was widely distributed at all stages, *Lactobacillus was* predominant before solid fermentation for 25 h, and *Streptococcus* was prevailing at P25, SP, and F7. At the species level (Figure 6B), *Lactobacillus amylolyticus* was identified as the predominant bacterial at WP (33.1%) and P12 (36.1%), but rapidly decreased and became a minor community member after incubating with *Mucor* for 25 h. The other two major bacterial communities at P12, *Weissella confusa* (28.1%) and *Leuconostoc lactis* (7.8%), also experienced a sharp decline in abundance at P25. The massive proliferation of *Acinetobacter baumannii* (6.2%), *Bacillus cereus* (17.6%), and *Streptococcus infantarius* (42.2%) at P25 might inhibit their growth. For salted phetz, *Staphylococcus condimenti* (18.2%) was assigned to the most abundant bacteria apart from *Streptococcus infantarius* (34.3%). The rapid growth of *Staphylococcus condimenti* might attribute to its perfect salt tolerance. For the ripening stages, it was interesting to find that *Leuconostoc citreum* kept growing during the whole ripening process, while the abundance of *Weissella confusa* and *Streptococcus infantarius* had steadily declined. This might be due to their different tolerance to the dressing mixture containing ethanol.

For fungal communities, *Trichosporon* was dominant at all stages. Before salt was added to molded pehtze at SP, its abundance kept increasing (Figure 6C). As the salt concentration reduced from SP to F7, *Trichosporon* showed upregulation, and then gradually descended after ripening in the dressing for 30 days. The pervious study also proved the decrease of *Trichosporon* during sufu ripening (Wei et al., 2021). During solid-state fermentation, *Actinomucor elegans* and *Kluyveromyces marxianus* were introduced to the community, with the abundance of 18.4 and 17.0% at P12, but a descending tendency later (Figure 6D). This phenomenon was consistent with previous studies that demonstrated salt could inhibit the growth of *Mucor* as well as other salt-sensitive microorganisms (Han et al., 2004a; Xie et al., 2018b; He and Chung, 2020).

Compared with previous studies in sufu, significant differences occurred in the microbial communities. The predominant genus such as *Debaryomyces*, *Candida*, and *Aspergillus* (Xu et al., 2020; Wei et al., 2021; Li et al., 2022) detected in previous studies appeared to be minor or undetected in this study. This might be originated from the difference in the starter culture and detection technology. Moreover, microorganisms obtained from the environment also made a great contribution to the microbial profile.

### 3.3. Core functional microorganisms related to formation of umami peptides

**FIGURE 7:**
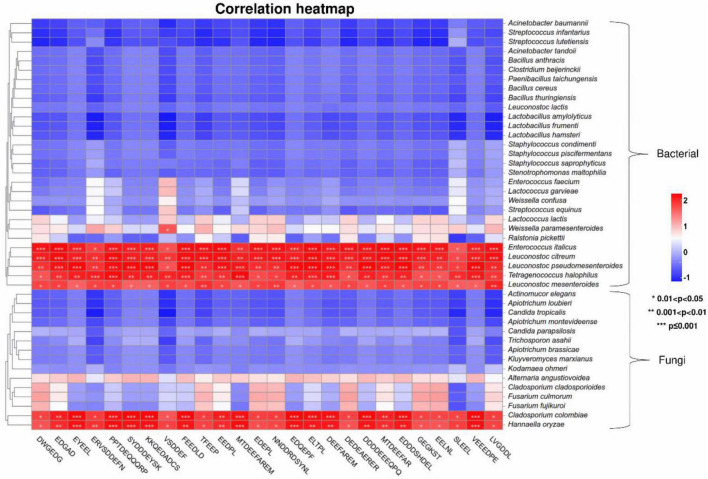
Clustering heatmaps based on Pearson’s correlation tests between the umami peptides and microorganisms based on species level.

**FIGURE 8:**
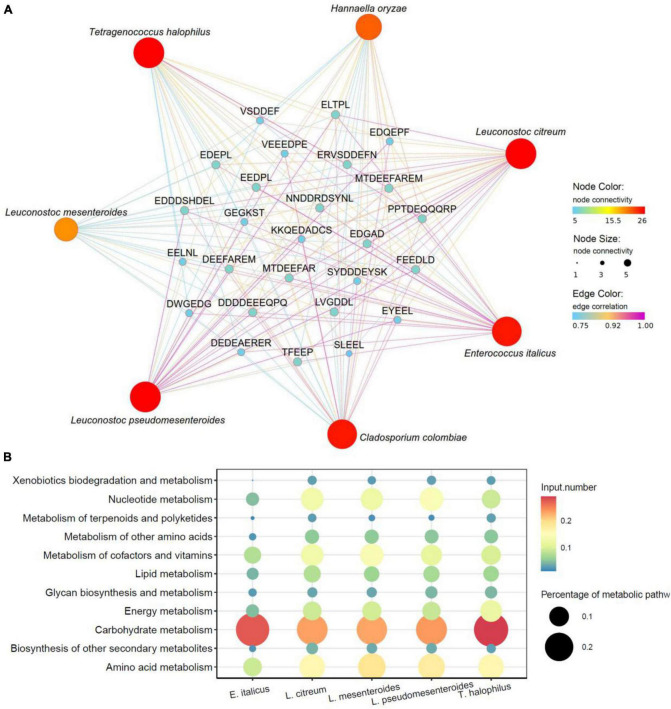
Correlation of microorganisms and peptides. **(A)** Correlation network maps based on Pearson’s correlation tests between the umami peptides and microorganisms based on species level. **(B)** KEGG enrichment analysis from *E. italicus*, *L. citreum*, *L. mesenteroides*, *L. pseudomesenteroides* and *T. halophilus*.

Microbial fermentation is one of the main production methods of soy peptides. As microorganisms carry out their own metabolic activities, mixed enzymes were produced to hydrolyze soy protein into peptides with different lengths and sequences (de Oliveira et al., 2015). Meanwhile, utilizing the nutrients in peptide hydrolyzate facilitates the growth and enzyme production capabilities of microorganisms, which significantly improves the hydrolysis efficiency (Cruz-Casas et al., 2021). In this study, the correlation between 26 umami peptides and identified microbial species including 29 bacterial and 15 fungi was analyzed using Pearson’s correlation tests. The core functional microorganisms related to the formation of umami peptides were selected based on the standard that average Pearson correlation coefficient *r* > 0.75 and *P* < 0.05. As shown in the correlation heatmap (Figure 7), 5 bacterial species and 2 fungi species were found significantly correlated with the 26 umami peptides. The correlation network was further analyzed in Figure 8. For fungi communities, only *Cladosporium Colombia* and *Hannaella oryzae* showed a significantly positive association with most of the umami peptides. However, their function on flavor and umami taste of fermented foods was seldom reported. For bacterial, the core functional genera related to umami peptides production were *Enterococcus italicus, Leuconostoc citreum*, *Leuconostoc mesenteroides*, *Leuconostoc pseudomesenteroides* and *Tetragenococcus halophilus*, which showed significant positive correlations with more than 20 umami peptides (Figure 8A). It was noticed that all five functional bacterial species belonged to lactic acid bacteria (LAB). They had been widely discovered from fermented foods such as cheese, kimchi, and soy sauce, and made great contributions to the flavor and taste (Nguyen et al., 2013). *Leuconostoc citreum* had been reported to exhibit glutaminase-producing activity in a traditional Thai fermented food (Thongsanit et al., 2009). *Tetragenococcus halophilus* was also proven to improve the flavor of fish sauce and increase the umami taste in soy sauce (Udomsil et al., 2011; Zhang et al., 2020). The genome sequences of five bacterial strains correlated with the formation of umami peptides were downloaded from NCBI. The specific information was shown in Supplementary Table 5. KEGG functional annotation was performed using eggNOG.^9^ The results showed that amino acid metabolism was the second important enrichment pathway (Figure 8B), which verified their umami peptides production ability.

## 4. Conclusion

Overall, this study clarified the changes in endogenous peptides, the functions of their precursor proteins, and the succession of microorganisms throughout the 8 stages of sufu fermentation. 26 novel umami peptides with increasing expression patterns were identified. Also, five lactic acid bacteria that facilitated umami peptides formation were discovered at the species level for the first time. Our results have certain guiding significance for the subsequent development and utilization of biological resources. By regulating the core functional microorganisms, it was possible to improve the flavor and quality of commercial sufu products. Also, the discovery of novel umami peptides might provide new ideas for the development of food seasonings in the condiment industry. Further studies should be performed to verify strain’s umami peptide producing ability through isolation and cultivation. The umami intensity of novel peptides should also be examined through sensory evaluation and electric tongue analysis.

## Data availability statement

The original contributions presented in this study are included in the article/Supplementary material, further inquiries can be directed to the corresponding author.

## Author contributions

JM: conceptualization, data curation, visualization, writing – original draft, and writing – review and editing. ZZ: resources and writing – review and editing. HY: supervision, methodology, writing – review and editing, and resources. All authors contributed to the article and approved the submitted version.

## Footnotes

1
https://www.ncbi.nlm.nih.gov/research/cog-project/

2
http://www.expasy.ch/sprot

3
http://www.genome.jp/kegg

4
http://www.geneontology.org/

5
http://camt.pythonanywhere.com/iUmami-SCM

6
http://pmlabstack.pythonanywhere.com/UMPred-FRL

7
http://tastepeptides-meta.com/cal

8
https://lima.how/

9
http://eggnog-mapper.embl.de/
