# Supplementary material for: Microbial succession and its effect on the formation of umami peptides during sufu fermentation
Source: Front Microbiol. 2023 Apr 17;14:1181588. doi: 10.3389/fmicb.2023.1181588 (PMC10149673; doi:10.3389/fmicb.2023.1181588)
Supplement: Supplementary file 1 [file Table_1.DOCX]

| **Table S1. GO functional enrichment analysis of the Precursor proteins for differential peptides (Qvalue < 0.05)** | | | | |
| --- | --- | --- | --- | --- |
| GO ID | class | Descrption | No. of precursor proteins | Qvalue |
| GO:0004197 | Biological Process | proteolysis involved in cellular protein catabolic process | 61 | 7.23945E-16 |
| GO:0008234 | Biological Process | cellular protein catabolic process | 61 | 7.23945E-16 |
| GO:0005764 | Biological Process | protein catabolic process | 63 | 1.485E-15 |
| GO:0004175 | Biological Process | organonitrogen compound catabolic process | 63 | 2.48186E-15 |
| GO:0051603 | Biological Process | cellular macromolecule catabolic process | 64 | 2.53886E-15 |
| GO:0044257 | Biological Process | macromolecule catabolic process | 79 | 6.88607E-15 |
| GO:0030163 | Biological Process | organic substance catabolic process | 98 | 1.45382E-13 |
| GO:0070011 | Biological Process | cellular catabolic process | 98 | 1.45382E-13 |
| GO:0008233 | Biological Process | catabolic process | 102 | 2.95714E-13 |
| GO:1901565 | Biological Process | proteolysis | 68 | 3.27488E-13 |
| GO:0044265 | Biological Process | primary metabolic process | 212 | 0.002634799 |
| GO:0005615 | Biological Process | organic substance metabolic process | 217 | 0.00553556 |
| GO:0044421 | Biological Process | cellular macromolecule metabolic process | 174 | 0.005654287 |
| GO:0009057 | Biological Process | macromolecule metabolic process | 178 | 0.00812054 |
| GO:0000323 | Biological Process | nitrogen compound metabolic process | 192 | 0.00947712 |
| GO:1901575 | Biological Process | metabolic process | 245 | 0.032770079 |
| GO:0044248 | Cellular Component | lysosome | 53 | 4.16922E-20 |
| GO:0009056 | Cellular Component | extracellular space | 49 | 2.8708E-15 |
| GO:0006508 | Cellular Component | extracellular region part | 49 | 2.8708E-15 |
| GO:0016787 | Cellular Component | lytic vacuole | 60 | 1.08585E-13 |
| GO:0005576 | Cellular Component | extracellular region | 87 | 6.96368E-06 |
| GO:0044238 | Cellular Component | vacuole | 158 | 0.01588923 |
| GO:0003824 | Molecular Function | cysteine-type endopeptidase activity | 54 | 2.05669E-20 |
| GO:0071704 | Molecular Function | cysteine-type peptidase activity | 54 | 2.05669E-20 |
| GO:0044260 | Molecular Function | endopeptidase activity | 54 | 2.06523E-17 |
| GO:0043170 | Molecular Function | peptidase activity, acting on L-amino acid peptides | 56 | 1.91022E-15 |

| **Table S2. KEGG functional enrichment analysis of the precursor proteins for differential peptides (Qvalue < 0.05)** | | | |
| --- | --- | --- | --- |
| KEGG_A_class | KEGG_B_class | Pathway | No. of precursor proteins |
| Metabolism | Global and overview maps | Carbon metabolism | 34 |
| Genetic Information Processing | Folding, sorting and degradation | Protein processing in endoplasmic reticulum | 31 |
| Metabolism | Carbohydrate metabolism | Glycolysis / Gluconeogenesis | 27 |
| Metabolism | Carbohydrate metabolism | Starch and sucrose metabolism | 21 |
| Metabolism | Global and overview maps | Biosynthesis of amino acids | 21 |
| Genetic Information Processing | Translation | Ribosome | 15 |
| Metabolism | Lipid metabolism | alpha-Linolenic acid metabolism | 13 |
| Metabolism | Carbohydrate metabolism | Glyoxylate and dicarboxylate metabolism | 12 |
| Genetic Information Processing | Transcription | Spliceosome | 12 |
| Metabolism | Metabolism of other amino acids | Glutathione metabolism | 10 |
| Metabolism | Amino acid metabolism | Tyrosine metabolism | 9 |
| Metabolism | Nucleotide metabolism | Purine metabolism | 9 |
| Metabolism | Amino acid metabolism | Arginine biosynthesis | 7 |
| Metabolism | Carbohydrate metabolism | Fructose and mannose metabolism | 7 |
| Metabolism | Lipid metabolism | Linoleic acid metabolism | 7 |
| Metabolism | Carbohydrate metabolism | Pyruvate metabolism | 7 |
| Metabolism | Amino acid metabolism | Cysteine and methionine metabolism | 7 |
| Cellular Processes | Transport and catabolism | Endocytosis | 7 |
| Genetic Information Processing | Translation | RNA transport | 7 |

| **Table S3. COG functional analysis of the precursor proteins for differential peptides** | | |
| --- | --- | --- |
| COG ID | No. of peptides | Description COG_category |
| O | 176 | Posttranslational modification, protein turnover, chaperones |
| S | 176 | Function unknown |
| E | 67 | Amino acid transport and metabolism |
| G | 45 | Carbohydrate transport and metabolism |
| Q | 39 | Secondary metabolites biosynthesis, transport and catabolism |
| T | 38 | Signal transduction mechanisms |
| J | 34 | Translation, ribosomal structure and biogenesis |
| K | 24 | Transcription |
| U | 21 | Intracellular trafficking, secretion, and vesicular transport |
| C | 19 | Energy production and conversion |
| A | 12 | RNA processing and modification |
| D | 12 | Cell cycle control, cell division, chromosome partitioning |
| F | 10 | Nucleotide transport and metabolism |
| P | 10 | Inorganic ion transport and metabolism |
| B | 9 | Chromatin structure and dynamics |
| Z | 9 | Cytoskeleton |
| L | 6 | Replication, recombination and repair |
| I | 5 | Lipid transport and metabolism |
| M | 4 | Cell wall/membrane/envelope biogenesis |
| H | 3 | Coenzyme transport and metabolism |
| V | 2 | Defense mechanisms |
| Y | 0 | Nuclear structure |
| N | 0 | Cell motility |
| W | 0 | Extracellular structures |
| R | 0 | General function prediction only |

| **Table S4. functional analysis of the precursor proteins of umami peptides** | |
| --- | --- |
| ID | Description COG_category |
| GEGKST | Formate--tetrahydrofolate ligase |
| TFEEP | Glycinin G4 |
| VSDDEF | Kunitz trypsin inhibitor |
| EEDPL | Phospholipase D |
| DEEFAREM | Lipoxygenase |
| EDGAD | DNA damage-binding protein 1 |
| EDEPL | Uncharacterized protein |
| FEEDLD | Formate dehydrogenase, mitochondrial |
| ELTPL | Malonyl-CoA:isoflavone 7-O-glucoside-6''-O-malonyltransferase |
| DWGEDG | 34 kDa maturing seed protein |
| NNDDRDSYNL | Beta-conglycinin alpha' subunit |
| SYDDDEYSK | Bowman-Birk type proteinase inhibitor D-II |
| EDDDSHDEL | Uncharacterized protein |
| DDDDEEEQPQ | Uncharacterized protein |
| LVGDDL | Phosphopyruvate hydratase |
| EELNL | PfkB domain-containing protein |
| ERVSDDEFN | Kunitz trypsin inhibitor |
| DEDEAERER | Uncharacterized protein |
| MTDEEFAREM | Lipoxygenase |
| VEEEDPE | Uncharacterized protein |
| KKQEDADCS | Tudor domain-containing protein |
| EDQEPF | Uncharacterized protein |
| EYEEL | Lipoxygenase |
| MTDEEFAR | Lipoxygenase |
| PPTDEQQQRP | Glycinin G1 |
| SLEEL | 60S ribosomal protein L13 |

| **Table S5. Genomic information of 5 representative strains of bacteria** | | | |
| --- | --- | --- | --- |
| species | strains | Genome size  /Mb | GenBank |
| *Enterococcus italicus* | DSM 15952 | 2.416 | GCA_000185365.1 |
| *Leuconostoc citreum* | CBA3621 | 1.903 | GCA_007954785.1 |
| *Leuconostoc mesenteroides* | SRCM102733 | 2.059 | GCA_009913915.1 |
| *Leuconostoc pseudomesenteroides* | FDAARGOS_1003 | 2.115 | GCA_016127255.1 |
| *Tetragenococcus halophilus* | MJ4 | 2.389 | GCA_001712815.1 |
